# Supplementary material for: Logistics of Bone Mineralization in the Chick Embryo Studied by 3D Cryo FIB‐SEM Imaging
Source: Adv Sci (Weinh). 2023 May 19;10(22):2301231. doi: 10.1002/advs.202301231 (PMC10401108; doi:10.1002/advs.202301231)
Supplement: Supplementary file 1 — Supporting Information [file ADVS-10-2301231-s001.pdf]

ADVANCED SCIENCE
Open Access

Supporting Information

for *Adv. Sci.*, DOI 10.1002/advs.202301231

Logistics of Bone Mineralization in the Chick Embryo Studied by 3D Cryo FIB-SEM Imaging

*Emeline Raguin, Richard Weinkamer, Clemens Schmitt, Luca Curcuraci and Peter Fratzl**

Supporting Information

Logistics of Bone Mineralization in the Chick Embryo Based on 3D Cryo FIB-SEM Imaging

*Emeline Raguin, Richard Weinkamer, Clemens Schmitt, Luca Curcuraci and Peter Fratzl**

Movie legends:

Movie S1: Perspective rendering of the images acquired in stack 2. The movie shows first, the processed mixed Inlens/SE images and then the BSE images. The stack highlights the 3D arrangement of the mineralized bone, the vesicles containing mineral precursors and the bone cells based on the segmentation of these structures. Pause 1: Enlarged processed image of the mixed Inlens/SE detectors taken from the acquisition plan (xy) showing the bone and the organic matrix. A nucleus is clearly identifiable. Pause 2: Enlarged processed image of the mixed Inlens/SE detectors taken from the reconstruction plan (yz) showing the bone and the organic matrix in a quality similar to the acquisition plan due to the isometric and high-resolution voxel size (8nm). Pause 3: Enlarged processed image of first the BSE detector showing the mineralized bone (yellow), the rough contour of the cells (dotted orange) and their nucleus when present (turquoise). The green arrows point to mineral precursors that appear brighter in the BSE images. The same image from the mixed Inlens/SE detector shows that the mineral precursors identified in the BSE image are found within numerous vesicles and can be identified with a granular structure. Unmineralized collagen fibrils can be seen between the cells and the mineralized bone.

Movie S2: The movie shows the same stack as in Movie 1 with the segmented data. The nanochannels (blue) form an interconnected network with the canaliculi (red) in the mineralized bone matrix (light yellow). Numerous vesicles (purple) containing mineral precursors (green) are found inside the cells (orange) close to the mineralized bone matrix.

Movie S3: Perspective rendering of a complete femur at EDD13, contrast-stained with phosphotungstic acid. The movie shows then the segmentation of the osteocyte lacunae performed on a region of interest of 1.3 mm in thickness, corresponding to a bone volume of 0.2028 mm^3. A total of 39 716 osteocytes lacunae were counted, which gives a density of 196 000 osteocytes lacunae/ mm^3.

**FIB SEM stack 1**								
Volume imaged	8nm voxel	4096*3072 store resolution	1625 images	32.77 µm x24.58µm x13 µm	10 471.33 μm^3			
**Segmentation**								
Mineralized bone	2734.51 μm^3							
Cells Total	4693.00 μm^3	Number of cells: 6						
Canaliculi	59.27 μm^3							
Nanochannels channels	398.11μm^3							
Total vesicles	72.71μm^3	Number of vesicles: 122	Minimum: 0.01 μm^3	Maximum: 3.47 μm^3	SD: 0.55 μm^3	Mean: 0.60 μm^3		
Total mineral precursors	7.11μm^3	Minimum: 0.001μm^3	Maximum: 0.45μm^3	SD: 0.07μm^3	Mean: 0.06μm^3			
Cell 1:	304.15μm^3	Volume Nucleus: 13.35 μm^3	Number of vesicles: 5	Total volume of vesicles: 2.75 μm^3	Minimum: 0.2 μm^3	Maximum: 1.08 μm^3	SD: 0.44 μm^3	Mean: 0.55 μm^3
				Total mineralization: 0.10 μm^3	Minimum: 0.002 μm^3	Maximum: 0.05 μm^3	SD: 0.02 μm^3	Mean: 0.02 μm^3
Cell 2:	296.95μm^3	Volume Nucleus: 9.15 μm^3	Number of vesicles: 6	Total volume of vesicles: 10.20 μm^3	Minimum: 0.66 μm^3	Maximum: 3.47 μm^3	SD:0.88 μm^3	Mean: 1.70 μm^3
				Total mineral precursors: 1.13 μm^3	Minimum: 0.06 μm^3	Maximum: 0.45 μm^3	SD: 0.13 μm^3	Mean: 0.19 μm^3
Cell 3:	469.13 μm^3	Volume Nucleus: 18.53 μm^3	No vesicles					
Cell 4:	212.46 μm^3	No nucleus	No vesicles					
Cell 5:	1445.69 μm^3	No nucleus	Number of vesicles: 32	Total volume of vesicles:	Minimum: 0.13 μm^3	Maximum: 1.69 μm^3	SD: 0.36 μm^3	Mean: 0.58 μm^3

				17.91 µm³				
				Total mineral precursors: 1.84 µm³	Minimum: 0.006 µm³	Maximum: 0.33 µm³	SD: 0.06 µm³	Mean: 0.06 µm³
Cell 6:	1905.67 µm³	Volume Nucleus: 43.21 µm³	Number of vesicles: 79	Total volume of vesicles: 41.86 µm³	Minimum: 0.02 µm³	Maximum: 2.70 µm³	SD: 0.49 µm³	Mean: 0.53 µm³
				Total mineral precursors: 4.03 µm³	Minimum: 0.001 µm³	Maximum: 0.32 µm³	SD: 0.07 µm³	Mean: 0.07 µm³
**FIB SEM stack 2**								
Volume imaged	8nm voxel	4096*3072 store resolution	1876 images	32.768µm x 24.576µm x 15.008µm	12 086.038 µm³			
**Segmentation**								
Mineralized bone	2626.39 µm³							
Cells Total	8164.10 µm³	Number of cells: 6						
Canaliculi	88.23 µm³							
Nanochannels channels	378.27 µm³							
Total vesicles	199.14 µm³	Number of vesicles: 270	Minimum: 0.03 µm³	Maximum: 5.14 µm³	SD: 0.66 µm³	Mean: 0.74 µm³		
Total mineral precursors	16.29 µm³	Minimum: 0.0004 µm³	Maximum: 0.64 µm³	SD: 0.07 µm³	Mean: 0.06 µm³			
Cell 1:	359.47 µm³	Volume Nucleus: 181.49 µm³	Number of vesicles: 2	Total volume of vesicles: 2.17 µm³	Minimum: 1.06 µm³	Maximum: 1.11 µm³	SD: 0.03 µm³	Mean: 1.09 µm³
				Total mineral precursors: 0.08 µm³	Minimum: 0.004 µm³	Maximum: 0.07 µm³	SD: 0.03 µm³	Mean: 0.04 µm³
Cell 2:	822.78 µm³	No nucleus	Number of vesicles: 14	Total volume of vesicles: 8.02 µm³	Minimum: 0.09 µm³	Maximum: 1.58 µm³	SD: 0.46 µm³	Mean: 0.57 µm³

				Total mineral precursors: 0.67 μm^3	Minimum: 0.03 μm^3	Maximum: 0.18 μm^3	SD: 0.06 μm^3	Mean: 0.06 μm^3
Cell 3:	1221.70 μm^3	Volume Nucleus: 57.91 μm^3	Number of vesicles: 29	Total volume of vesicles: 37.40 μm^3	Minimum: 0.05 μm^3	Maximum: 5.14 μm^3	SD: 1.19 μm^3	Mean: 1.29 μm^3
				Total mineral precursors: 1.46 μm^3	Minimum: 0.01 μm^3	Maximum: 0.22 μm^3	SD: 0.06 μm^3	Mean: 0.05 μm^3
Cell 4:	134.48 μm^3	No nucleus	Number of vesicles: 1	Total volume of vesicles: 1.40 μm^3				
				Total mineral precursors: 0.01 μm^3				
Cell 5:	3564.34 μm^3	Volume Nucleus: 119.49 μm^3	Number of vesicles: 146	Total volume of vesicles: 106.30 μm^3	Minimum: 0.03 μm^3	Maximum: 3.25 μm^3	SD: 0.57 μm^3	Mean: 0.73 μm^3
				Total mineral precursors: 9.95 μm^3	Minimum: 0.0004 μm^3	Maximum: 0.64 μm^3	SD: 0.08 μm^3	Mean: 0.08 μm^3
Cell 6:	2064.36 μm^3	Volume Nucleus: 119.08 μm^3	Number of vesicles: 78	Total volume of vesicles: 43.85 μm^3	Minimum: 0.07 μm^3	Maximum: 2.90 μm^3	SD: 0.41 μm^3	Mean: 0.56 μm^3
				Total mineral precursors: 4.10 μm^3	Minimum: 0.0004 μm^3	Maximum: 0.29 μm^3	SD: 0.06 μm^3	Mean: 0.06 μm^3
**FIB SEM stack 3**								
Volume imaged	8nm voxel	4096*2460 store resolution	303 images	32.77 µm x19.68 µm x2.424 µm	1563.3 μm^3			
**Segmentation**								
Mineralized bone	767.87 μm^3							

Cells Total	473.49 µm³	Number of cells: 7						
Canaliculi	7.93 µm³							
Nanochannels channels	109.39 µm³							
Total vesicles	10.20 µm³	Number of vesicles: 40	Minimum: 0.002 µm³	Maximum: 1.04 µm³	SD: 0.25 µm³	Mean: 0.24 µm³		
Total mineral precursors	1.01 µm³	Minimum: 0.001 µm³	Maximum: 0.21 µm³	SD: 0.004 µm³	Mean: 0.02 µm³			
Osteocyte 1:	114.44 µm³	Volume Nucleus: 48.24 µm³	Number of vesicles: 13	Total volume of vesicles: 3.25 µm³	Minimum: 0.04 µm³	Maximum: 0.68 µm³	SD: 0.18 µm³	Mean: 0.27 µm³
				Total mineral precursors: 0.25 µm³	Minimum: 0.002 µm³	Maximum: 0.05 µm³	SD: 0.01 µm³	Mean: 0.02 µm³
Osteocyte 2:	86.72 µm³	Volume Nucleus: 29.37 µm³	Number of vesicles: 9	Total volume of vesicles: 2.25 µm³	Minimum: 0.05 µm³	Maximum: 1.04 µm³	SD: 0.37 µm³	Mean: 0.34 µm³
				Total mineral precursors: 0.20 µm³	Minimum: 0.001 µm³	Maximum: 0.10 µm³	SD: 0.03 µm³	Mean: 0.03 µm³
Osteocyte 3:	27.53 µm³	No nucleus imaged	No vesicles inside					
Cell 4:	111.04 µm³	Volume Nucleus: 68.18 µm³	Number of vesicles: 3	Total volume of vesicles: 1.27 µm³	Minimum: 0.19 µm³	Maximum: 0.84 µm³	SD: 0.30 µm³	Mean: 0.42 µm³
				Total mineral precursors: 0.02 µm³	Minimum: 0.01 µm³	Maximum: 0.01 µm³	SD: 0.001 µm³	Mean: 0.01 µm³
Cell 5:	45.79 µm³	Volume Nucleus: 9.83 µm³	Number of vesicles: 7	Total volume of vesicles: 2.48 µm³	Minimum: 0.06 µm³	Maximum: 0.91 µm³	SD: 0.30 µm³	Mean: 0.35 µm³

				Total mineral precursors: 0.44 µm³	Minimum: 0.01 µm³	Maximum: 0.21 µm³	SD: 0.06 µm³	Mean: 0.06 µm³
Cell 6:	66.19 µm³	No nucleus imaged	Number of vesicles: 6	Total volume of vesicles: 0.8 µm³	Minimum: 0.01 µm³	Maximum: 0.40 µm³	SD: 0.12 µm³	Mean: 0.15 µm³
				Total mineral precursors: 0.07 µm³	Minimum: 0.001 µm³	Maximum: 0.03 µm³	SD: 0.01 µm³	Mean: 0.01 µm³
Cell 7:	21.78 µm³	No nucleus imaged	Number of vesicles: 2	Total volume of vesicles: 0.13 µm³	Minimum: 0.06 µm³	Maximum: 0.08 µm³	SD: 0.01 µm³	Mean: 0.07 µm³
				Total mineral precursors: 0.02 µm³	Minimum: 0.01 µm³	Maximum: 0.01 µm³	SD: 0.003 µm³	Mean: 0.01 µm³

Table S1: Raw quantitative data for all three stacks investigated. All volumes and counts are based on the segmentation of the different structures using Dragonfly software (Object Research Systems (ORS) Inc, Montreal, Canada).

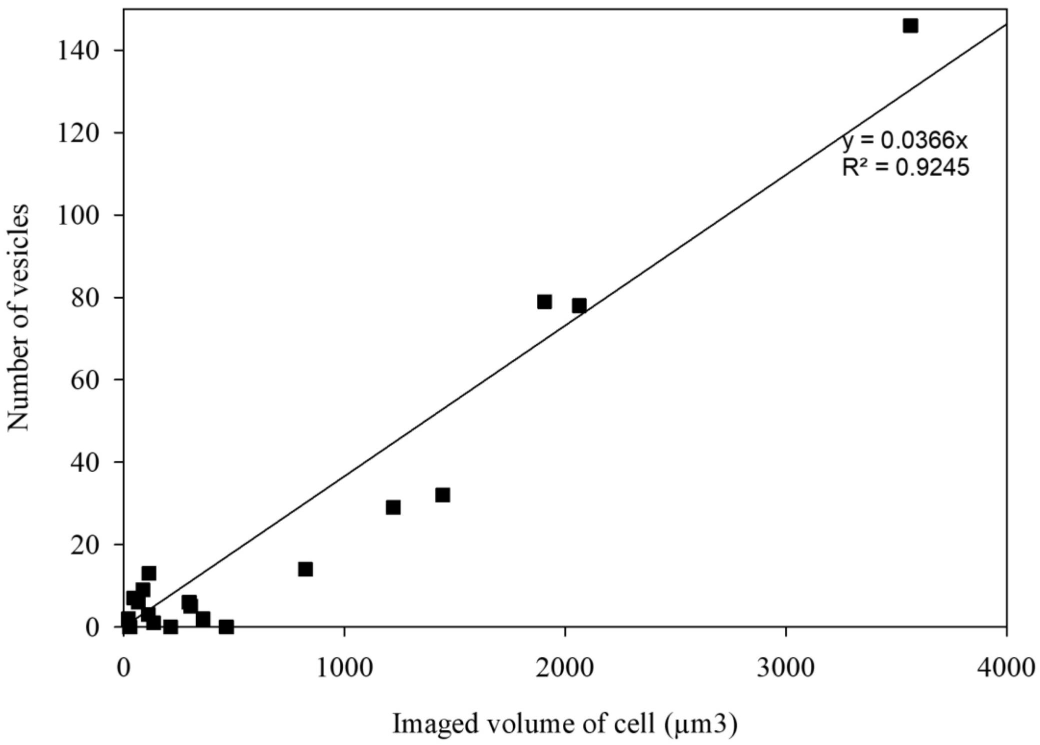

Figure S1: Linear regression of the number of vesicles according to the volume of cell partially imaged for the three stack combined. The pre-factor of 0.0366 correspond to the vesicles density.
